# On the Restriction and Prevention of Tuberculosis

**Published:** 1894-04

**Authors:** 


### Selections and Abstracts.

---

On the Restriction and Prevention of Tuberculosis.—The committee appointed by the American Public Health Association, in conjunction with the International Congress of Public Health, has published the following report:

1. Tuberculosis has been conclusively demonstrated to be contagious, by bacteriological experiments, by clinical observations, and by a study of the history of the disease.

2. Tuberculosis is a preventable disease. Its preventability follows as a logical sequence upon its contagiousness, but has likewise been demonstrated in a practical life.

3. The contagium of tuberculosis resides entirely in broken-down tubercular tissue. A person suffering from tuberculosis, therefore, does not become a source of danger to others until he begins to give off broken-down tubercular tissue, either in the form of sputa from the throat or lungs, diarrheal discharges from the bowels, or matter from a tuberculous sore, such as lupus, white swelling, cold, abscess, scrofula, or tubercular inflammation of a joint.

4. A person suffering from tuberculosis can be made entirely harmless to those about him by thorough sterilization of all broken-down tissue, immediately upon its being given off. With proper precautions it is, therefore, possible to live in the closest relationship, and upon the most intimate terms, with consumptives, without contracting the disease.

5. Tuberculosis is not hereditary. A predisposition to the disease can be transmitted from parent to offspring, but this is more true of tuberculosis than it is of all other contagious diseases.

6. A predisposition to tuberculosis can be created anew by malnutrition, or by anything which depresses the nervous system.

7. Tuberculosis affects animals as well as man, and is identically the same disease in both. In domestic life human beings and animals mutually infect each other.

8. The media through which human beings are ordinarily infected by animals are milk and meat.

9. Houses in which consumptives have lived, and in which immediate sterilization of all broken-down tissue has not been practiced, are infected houses, and are liable to convey the disease to subsequent occupants.

10. Spitting upon floors and into handkerchiefs, and permitting the broken-down tissue to dry and become pulverized, is a prolific cause of spreading tuberculosis.

11. Temporary occupation of hotel rooms, sleeping car berths, and steamer cabins by consumptives in the infectious state can infect them so as to convey the disease to subsequent occupants, unless proper precautions are taken against contamination of the bedding, furniture and walls with broken-down tubercular tissue.

We recommend the following practical measures for the prevention of the disease:

1. The notification and registration by health authorities of all cases of tuberculosis which have arrived at the infectious stage.

2. The thorough disinfection of all houses in which tuberculosis has occurred, and the recording of such action in open record.

3. The establishment of special hospitals for the prevention of tuberculosis.

4. The organization of societies for the prevention of tuberculosis.

5. Goverment inspection of dairies and slaughter houses, and the extermination of tuberculosis among dairy cattle.

6. Appropriate legislation against spitting into places where the sputum is liable to infect others, and against the sale or donation of objects which have been in use by consumptives, unless they have been thoroughly disinfected.

7. Compulsory disinfection of hotel rooms, sleeping car berths, and steamer cabins which have been occupied by consumptives, before other persons are allowed to occupy them.—*N. Y. Med. Record.*

